# Early Canine Plaque Biofilms: Characterization of Key Bacterial Interactions Involved in Initial Colonization of Enamel

**DOI:** 10.1371/journal.pone.0113744

**Published:** 2014-12-02

**Authors:** Lucy J. Holcombe, Niran Patel, Alison Colyer, Oliver Deusch, Ciaran O’Flynn, Stephen Harris

**Affiliations:** The WALTHAM Centre for Pet Nutrition, Leicestershire, United Kingdom; University Hospital of the Albert-Ludwigs-University Freiburg, Germany

## Abstract

Periodontal disease (PD) is a significant problem in dogs affecting between 44% and 63.6% of the population. The main etiological agent for PD is plaque, a microbial biofilm that colonizes teeth and causes inflammation of the gingiva. Understanding how this biofilm initiates on the tooth surface is of central importance in developing interventions against PD. Although the stages of plaque development on human teeth have been well characterized little is known about how canine plaque develops. Recent studies of the canine oral microbiome have revealed distinct differences between the canine and human oral environments and the bacterial communities they support, particularly with respect to healthy plaque. These differences mean knowledge about the nature of plaque formation in humans may not be directly translatable to dogs. The aim of this study was to identify the bacterial species important in the early stages of canine plaque formation *in vivo* and then use isolates of these species in a laboratory biofilm model to develop an understanding of the sequential processes which take place during the initial colonization of enamel. Supra-gingival plaque samples were collected from 12 dogs at 24 and 48 hour time points following a full mouth descale and polish. Pyrosequencing of the 16S rDNA identified 134 operational taxonomic units after statistical analysis. The species with the highest relative abundance were *Bergeyella zoohelcum*, *Neisseria shayeganii* and a *Moraxella* species. Streptococcal species, which tend to dominate early human plaque biofilms, had very low relative abundance. *In vitro* testing of biofilm formation identified five primary colonizer species, three of which belonged to the genus *Neisseria*. Using these pioneer bacteria as a starting point, viable two and three species communities were developed. Combining *in vivo* and *in vitro* data has led us to construct novel models of how the early canine plaque biofilm develops.

## Introduction

Periodontitis is one of the most prevalent diseases in both humans and dogs. In both species, studies have estimated that approximately 45% to 65% of the population are affected by the condition with rates varying depending on age, diagnosis criteria and also, in the case of dogs, breed [1]–[5]. Several decades of work on human dental plaque has led to an understanding of many of the ecological processes important in the initiation and maturation of these polymicrobial biofilm communities, yet the specifics of disease development remain unclear [6]–[8]. Our knowledge of the process in other systems is much less developed. Dogs represent an enticing study opportunity in this respect not simply as a comparison to human health but as a direct application to a health issue in this species. The diversity of canine oral microbiota has been well characterized using culture-independent methods and the associations of specific bacteria with health or early disease are understood [9]–[11]. Furthermore, the bacterial population in canine plaque was shown to be widely divergent from that of humans with only 16.4% of taxa shared [10]. This suggests that during canine oral biofilm development there may be alternative mechanisms at play driving microbial succession and, as a result, the progression of periodontitis. Studying how human and canine PD develop in response to differing microbial communities may in time allow shared, and therefore important, features in the microbial succession process to be identified.

Typically, colonization of a fresh oral surface follows a unidirectional pattern, meaning that only certain bacteria have the ability to bind at the outset and initiate biofilm formation, while the majority require the environment to have been prepared for them in some way by other species. In humans bacteria of the genus *Streptococcus* are typically reported to fulfil this primary colonization role, binding to the salivary pellicle in a short time frame and paving the way for the recruitment of successive species [12]. A conceptual spatiotemporal model of human plaque biofilm formation, based on accumulated data from the literature, was developed by Socransky and co-workers in 1998 [6] and has become the accepted paradigm for plaque colonization. The model supports the long held view that the transition from health to disease is characterized by a switch from the presence of mainly Gram positive species to mainly Gram negative species [13]. Although some recent studies have suggested this may be an over-simplification of the real-life environment [14], [15] many of the early colonizer bacteria identified support the theory. The discovery that not only is this situation reversed in canine plaque (with health associated species mostly falling into the Gram negative classification) but also that there is a distinct lack of streptococcal species [11], suggests that any extrapolation from the human model to the oral colonization process in dogs is likely to be inaccurate.

While an investigation of the interactions between canine bacterial species (cultured isolates from plaque and saliva) using a co-aggregation assay has previously been described [16] the recent advances in the characterization of the canine oral microbiome, along with the advent of sophisticated and accurate molecular identification techniques, means it is now possible to explore early canine plaque formation in detail.

The aim of this study was to enhance our developing picture of the microbiology of canine plaque, providing an understanding of the bacterial species and interactions important in early biofilm formation. Initially nascent communities were studied *in vivo*, using pyrosequencing to characterize the biofilm microbiota in supra-gingival plaque. Species-specific qPCR assays were then used to define binding interactions of key isolates involved in early biofilm formation, allowing insights into the complex relationships that exist between bacterial species in canine oral biofilms to be explored.

## Materials and Methods

### Ethical statement and sample collection

Sample collections described in this study were approved by The WALTHAM Centre for Pet Nutrition Animal Welfare and Ethical Review Body. The dogs were owned by WALTHAM and were housed at WALTHAM in kennels that exceeded the requirements of the Animal (Scientific Procedures) Act 1986 Code of Practice. Only dogs anaesthetized (propofol, 4 mg kg^−1^) for routine dental treatment as part of their annual health assessment performed by the site veterinarian were used for sampling. Supra-gingival plaque was collected from Labrador retriever dogs (8 female, 4 male) at 24 and 48 hour time points from a clean-mouth model starting point. The mean age of the dogs was 4 years ±0.9 (SD). The periodontal health status of each dog was recorded using the Lobprise and Wiggs scoring system [17] at the time of the initial dental treatment. Plaque was collected from the outer buccal tooth surfaces using sterile 1 µL plastic loops. Samples from one half of the mouth (teeth 101–109 and 401–410) were collected at 24 hours post descale and polish and samples from the other half of the mouth collected at 48 hours post descale and polish (teeth 201–209 and 301–310). Tooth-brushing, which would normally be performed following a routine scale and polish was suspended during this 48 hour period. Plaque filled loop ends were collected into 300 µL Tris-EDTA (TE) buffer (pH 8.0) in a 2 mL microcentrifuge tube (one tube per dog per time-point) and vortexed vigorously. Samples were stored at −80°C prior to the extraction of DNA. Stimulated, whole mouth, canine saliva was collected from trained dogs. Saliva was collected onto cotton wool swabs which were transferred to Salivette collection tubes (Sarstedt, Germany). Saliva was then eluted from the swabs by centrifugation at 1000×*g* for 5 minutes at 4°C. Samples from each dog were pooled, filtered through a 0.22 µM filter to remove bacteria and stored at −20°C.

### DNA extraction and 16S rDNA amplification

Microbial genomic DNA was extracted using the Epicentre Masterpure Gram Positive DNA Extraction kit according to the manufacturer’s instructions for bacteria (Epicentre, USA). An extended overnight lysis step was used whereby 1 µL Ready-Lyse Lysozyme (Epicentre, USA) was added to each sample and the mixture incubated at 37°C for 18 hours. The final purified genomic DNA pellet was suspended in 35 µL TE buffer (pH 8.0) and quantified using a Qubit fluorometer (Life Technologies, USA) with the manufacturer’s dsDNA High Sensitivity (HS) assay. The 16S rDNA was amplified for sequencing using primers designed to the V1–V3 variable region. A mastermix was prepared comprising Extensor Hi-Fidelity PCR Enzyme Mix (Thermo, UK) and a mixture of two universal forward primers; FLX_27FYM (*CGTATCGCCTCCCTCGCGCCATCAG* **AGAGTTTGATYMTGGCTCAG**) used at 9.5 pmol µL^−1^ and FLX_27F_Bif (*CGTATCGCCTCCCTCGCGCCATCAG* **AGGGTTCGATTCTGGCTCAG** ) **used at 0.5 pmol µL^−1^ (where italics represent FLX Titanium Primer A and bold represents 16S rDNA primer sequence). The FLX_27F_Bif primer was included to ensure representation of the genus *Bifidobacter*, which is not amplified well by FLX_27FYM; a lower concentration was used due to the reduced representation of this genus identified in previous studies of canine plaque [11]. The reverse primer, used at 10 pmol µL^−1^, had 1 of 20 different 7mer MID tags incorporated;**

(***CTATGCGCCTTGCCAGCCCGCTCAG*XXXXXXX****TYACCGCGGCTGCTGG) **where italics represent FLX Titanium Primer B, X represents MID sequence and bold represents 16S rDNA reverse primer sequence. For PCR the cycling conditions were as follows; 94°C for 3 minutes; 10 cycles of 94°C for 45 seconds, 55°C for 30 s and 72°C for 60 s; followed by a further 20 cycles of 94°C for 45 s, 55°C for 30 s and 72°C for 90 s and a final extension of 72°C for 5∶30 minutes.**

### Library preparation

Purification of PCR products, library preparation, emulsion PCR and sequencing of the 16S rDNA amplicons were carried out by Eurofins MWG Operon (Germany) according to Roche’s amplicon library protocol. Samples were pooled into equimolar groups of 20 prior to Emulsion PCR. Libraries were sequenced on a Roche Genome Sequencer Junior system using the FLX Titanium B primer only, with a target of 7,500 unidirectional reads per sample.

### Sequence processing and analysis

Sequence data has been uploaded to the European Bioinformatics Institute (EBI) short read archive under study accession PRJEB7013. The standard flowgram files (SFF) were initially filtered by selecting reads with a minimum of 360 flows and truncating after a maximum of 720 flows. Reads were filtered and denoised using the AmpliconNoise software (version v1.21 [18], [19]). For the initial filtering step, reads were truncated when flow signals dropped below 0.7, indicative of poor quality. A maximum of 20,000 reads per sample was used due to the computational demands of the denoising algorithm. Subsequently reads were denoised in three stages: 1) Pyronoise to remove 454 sequencing errors (PyronoiseM parameters -s 60, -c 0.01), followed by truncation after 350 nucleotides; 2) Seqnoise to remove errors resulting from PCR amplification (SeqNoiseM parameters -s 25, -c 0.08); 3) Perseus to detect and remove chimeras resulting from PCR recombination. Sequences were then inflated so that all reads of the same sequence are represented by separate sequences in FASTA format. Denoised sequences were clustered using QIIME [20], a pipeline for performing microbial community analysis. The QIIME script pick_otus.py and UCLUST (version v.1.2.22q [21]) were used to cluster sequences with >98% identity. UCLUST was run with modified parameters, with gap opening penalty set to 2.0 and gap extension penalty set to 1.0 and –A flag to ensure optimal alignment. The most abundant sequence was picked as the representative sequence of each cluster. Representative sequences of all observed operational taxonomic units (OTUs) that passed the filtering criteria for sequence abundance (see statistical analysis section) were annotated using BLASTn of NCBI-BLAST 2.2.27+ [22] against the Canine Oral Microbiome Database (COMD) 13, which contains 416 published 16S sequences obtained from canine oral taxa (Genbank accession numbers JN713151–JN713566 [10]). Representative sequences were additionally annotated against the Silva SSU database release 115 [23], [24] which contains 479,726 sequences. For each representative sequence the best BLAST hit in the COMD database was chosen as the reference sequence. Cut-offs of 98.5% were applied for identity and coverage (alignment length divided by query length) for annotation to species level, if the alignment did not meet the cut-off criteria the best hit from the Silva database was chosen. Where species level annotation was not possible identity cut-offs of >95% were used to define OTUs to genus level or >90% to family level.

#### Visualization of the most abundant OTUs

OTUs with at least 1% average abundance at either time point were selected for visualization. Full-length reference sequences for the resulting 22 OTUs were extracted from COMD 13 (20 OTUs) and Silva SSU database release 115 (OTUs 21580 and 2868) and aligned using MUSCLE v3.8.31 [25]. An approximate maximum likelihood tree was inferred using FastTree v2.1.7 [26] using a generalized time-reversible model. The relative abundance bubble plot was created using the R package ggplot2.

### Bacterial culture conditions and biofilm formation

Bacterial strains (see Table S1) were isolated from canine plaque samples and grown in supplemented Brain Heart Infusion (BHI) broth at 38°C. Strains preferring microaerophilic growth conditions were incubated in nitrogen containing 5% O_2_ and 10% CO_2_ in a MG1000 Anaerobic Work Station (Don Whitley Scientific Ltd., United Kingdom). Taxonomy of all natural isolates was routinely confirmed by full-length 16S rRNA Sanger sequencing using ‘universal’ degenerate primers 8F (AGAGTTTGATYMTGGCTCAG) and C72 (GYTACCTTGTTACGACTT). Sequence identities were confirmed against the COMD (10). Cell pellets were washed three times in Dulbecco’s Phosphate Buffered Saline (DPBS) before being resuspended in 25%, filter-sterilized (0.2 µM), pooled canine saliva at a cell density of approximately 1 × 10^6^ cells ml^−1^. The bacteria-saliva suspensions were added in 1 mL aliquots to individual 5 mm×2 mm hydroxyapatite (HA) discs (Clarkson Chromatography, USA) which had been primed in 100% filter-sterilized canine saliva for 1 hour at 38°C to allow formation of a salivary pellicle. After incubation at 38°C for 2 hours, to allow for bacterial binding to the disc, the bacterial suspension was removed and the disc washed once in sterile DPBS to remove any unbound bacteria. Fresh filter-sterilized 25% saliva was added to the wells (1 mL) and the plate returned to the incubator for a further 24 hours. Incubation was stopped by removal of saliva and washing of the disc once using DPBS. Biofilm formation was assessed visually using Crystal violet stain (Sigma, UK) and Alamar blue (resazurin) stains (Sigma, UK). Crystal violet incorporates into bacterial surface molecules and is used to indicate the amount of bacteria bound to the HA surface [27], [28]. Alamar blue, otherwise known as resazurin, which is converted to pink resorufin in the presence of metabolically active cells was included to provide information on the health of biofilms [28], . For the Crystal violet assay HA-bound bacteria were fixed onto discs by incubation in 100 µL methanol for 15 minutes. The methanol was then removed and discs were air-dried for 20 minutes before incubation with Crystal violet for 20 minutes at room temperature. Residual stain was removed by 3 washes with distilled water. For the Alamar blue (resazurin) assay replicate biofilms, grown from the same bacterial culture, were incubated with 100 µL of 10 µg mL^−1^ Alamar blue (resazurin) stain at 38°C for 1 hour. Relative biofilm formation was ranked using a 4 point scale with the following categories: (−) no evidence of stained bacteria on disc with Crystal violet, Alamar blue solution does not change colour, (+) disc stained light blue or has light punctate spots with Crystal violet stain, purple color develops with Alamar blue stain, (++) dark punctate spots or blue disc with Crystal violet stain, light pink color with Alamar blue stain, (+++) strong dark blue staining over entire disc with Crystal violet stain, deep pink color with Alamar blue stain. Experiments were repeated in biological triplicate for each strain and negative control discs (saliva only) were included.

Additional biofilms grown as described above were used for molecular analysis. After conclusion of the incubation phase the HA disc was transferred to a 2 mL microfuge tube containing 300 µL TE buffer and vortex mixed vigorously for 2 minutes to remove the biofilm from the disc surface. DNA was extracted using the Epicentre Masterpure Gram Positive DNA Extraction kit as described above with a modification to include 2 µL of lysozyme for the overnight incubation step to account for the double volume of TE required to ensure complete coverage of the HA disc. HA discs were removed at the protein precipitation stage of the extraction.

For two and three species community tests equal amounts of bacteria were mixed and incubated for 4 hours with salivary pellicle coated discs before unbound bacteria were removed by washing with DPBS. Discs were then re-incubated with fresh filter-sterilized canine saliva (25%) at 38°C for a further 24 hours to allow growth of HA-bound bacteria. Biofilms formed were processed as described for primary colonizer experiments.

### Quantitative PCR (qPCR) analysis

All Taqman FAM-MGB bacterial 16S qPCR assays developed for this study (see Table S2) were either designed in house or by Primer Design Ltd. (UK). Each assay was tested for specificity against 16S rRNA clones representing the majority of taxa identified in the canine oral microbiome [11]; to pass specificity testing a 15 Cq difference between the target and any cross-reacting species was required. Clones were serially diluted for determination of assay efficiency and the LOQ. Efficiency was required to exceed 80% and the LOQ was defined as the highest dilution at which all triplicates were no longer within 0.25 Cq of each other. For a subset of assays the LOQ was checked against a background of a large excess of non-target plaque DNA, in all cases this comparison showed less than 7% difference in efficiency and 2 Cq change in LOQ between the target clone and the target spiked into plaque. The high level of similarity in the 16S rRNA sequence of *Neisseria* species meant it was not possible to design qPCR assays specific to each individual isolate. As a result a single assay in which primers can bind to DNA from COT-349, COT-090 and COT-269 was used. Each individual 10 µL qPCR reaction contained: 5 µL Applied Biosytems Gene Expression Taqman MasterMix (Applied Biosystems, USA); 0.5 µL 20X concentrated assay; 1 µL DNA and 3.5 µL nuclease-free water. Each assay contained a final concentration of 900 nM of each primer and 250 nM of each probe per qPCR reaction. Experiments were performed in triplicate with a positive control sample included in each run. This control sample contained a spike of clones representing targets for each of the assays used. Data were collected on an AB7900 HT machine (Applied Biosystems, USA) and analyzed using GenEx software (MultiD, Sweden). The analysis included; removal of samples outside of the Cq cut-off, removal of technical outliers (confidence level 0.95), assay efficiency correction and averaging of qPCR repeats. To assess bacterial growth Cq values were subtracted from the limit of quantification (LOQ) of the species-specific assay (see Table S2) and the mean Cq difference then converted to a fold change. This reports the amount of DNA present in the sample relative to a situation where so little DNA is present it is no longer possible to detect and quantify it accurately using the assay. As such it is a measure of presence/absence of bacteria rather than relative abundance.

### Statistical analysis

#### *In vivo* biofilms

OTUs were analyzed if they had at least one of the 24 hour or 48 hour averages greater than 0.05% and that average was due to presence in at least two dogs. The remaining OTUs were classed as ‘rare’ and grouped as such. The 0.05% cut-off was based on statistical analysis of data from mock communities containing 17 known species sequenced on five separate 454 runs [11]. To investigate the impact of time on OTU relative abundance generalized linear mixed model (GLMM) analyses were used for proportions (with binomial distribution and logit link) and also to allow for repeated sampling points on individual dogs. Time was fitted as a fixed effect and dog as a random effect. To allow the methods to enable robust estimation at very low proportions two counts were added to each bacterial count, and four counts were added to the total count (analogous to adding two successes and two failures [30]). The analysis of large numbers of OTUs increases the likelihood of obtaining false positives. To adjust for this multiplicity effect *p*-values were adjusted according to the false discovery method of Benjamini and Hochberg (BH) [31] to the 5% level.

#### Shannon diversity index

Shannon diversity indices were calculated for each sample [32] and analyzed by a linear mixed model, with dog as a random effect and time as a fixed effect. A test level of 5% was used.

#### qPCR primary colonizer assay

For identification of primary colonizers, qPCR log_10_ fold change (above the LOQ) values were analyzed by one-way ANOVA, with isolate strain as a fixed effect. The average log_10_ fold change values were compared between isolates using Tukey’s honestly significantly different (HSD) procedure, with homogeneous groups presented to the 5% test level.

## Results

### Species composition and characteristics of early canine oral biofilm communities *in vivo*

Early supra-gingival plaque biofilm communities were sampled 24 and 48 hours after professional teeth cleaning and analyzed by 454-pyrosequencing. The oral health status of each of the dogs was assessed during the dental procedure; all twelve dogs were classified as having mild or very mild gingivitis.

In total 173,642 quality sequences were generated and assigned to 3,100 operational taxonomic units (OTUs) using UCLUST and a cut-off of ≥ 98% sequence identity. The total number of sequences per sample ranged from 4,258 to 10,211 with a median number of reads of 6,924 in the 24 hour samples and 7,484 in the 48 hour samples. Statistical analysis resulted in 134 OTUs plus the rare group, with the rare group accounting for an average of 4.8% of sequence reads. Across all samples (both time points) 17 OTUs were present at ≥ 1% and these accounted for an average of 71.8% of sequence reads (Table 1). At the phylum level Proteobacteria were on average the most dominant (32.1%) followed by Bacteroidetes/Chlorobi (23.9%), Firmicutes (20.9%), Actinobacteria (14.9%) and Fusobacteria (6.0%). The remaining 2.2% belonged to the candidate phyla SR1 and TM7. The taxon with the highest relative abundance was *Bergeyella zoohelcum* (OTU #2184) accounting for 16.97% of the total sequence reads. This was followed by *Neisseria shayeganii* (OTU #637) and *Moraxella sp*. Canine Oral Taxon (COT) 017 (OTU #683), representing 10.41% and 9.31% of the sequence reads respectively (Table 1). In total, OTUs mapping to the genera *Neisseria* made up over 16% of the population. As a result, more than a third of the average early biofilm was accounted for solely by species belonging to the genera *Neisseria* and *Bergeyella*.

**Table 1:** Operational taxonomic units (OTUs) present at >1% of total sequence reads in supra-gingival plaque isolated 24 hour and 48 hours post de-scale and polish.

OTU	Species	Percentidentity	Percentcoverage	Total number ofsequence reads	Percentage of totalsequence reads
2184	Bergeyella zoohelcum	99.14	100	29467	16.97
637	Neisseria shayeganii	98.57	100	18068	10.41
683	Moraxella sp. COT-017	100	100	16167	9.31
991	Pasteurellaceae bacterium COT-080	100	100	11742	6.76
2629	Porphyromonas cangingivalis	99.71	100	9746	5.61
613	Neisseria weaveri	100	100	7862	4.53
2795	Capnocytophaga sp. COT-339	100	100	4951	2.85
163	Fusobacterium sp. COT-189	99.14	100	4096	2.36
2083	Pasteurella dagmatis	100	100	3447	1.99
580*	Actinomyces sp.	100	100	2902	1.67
1122	Lautropia sp. COT-175	99.71	100	2842	1.64
1366	Capnocytophaga cynodegmi	100	100	2593	1.49
326	Neisseria zoodegmatis	99.71	100	2588	1.49
21*	Corynebacterium sp.	100	100	2526	1.45
2160	Stenotrophomonas sp. COT-224	98.58	100	1960	1.13
234	Globicatella sp. COT-107	100	100	1948	1.12
226	Capnocytophaga canimorsus	100	100	1768	1.02

Cut-offs of 98.5% were applied for identity and coverage (alignment length divided by query length) for annotation to species level, if the alignment did not meet the cut-off criteria the best hit from the Silva database was chosen (indicated by *).

### Biofilm community composition shifts during early stages of development

**Figure 1:**
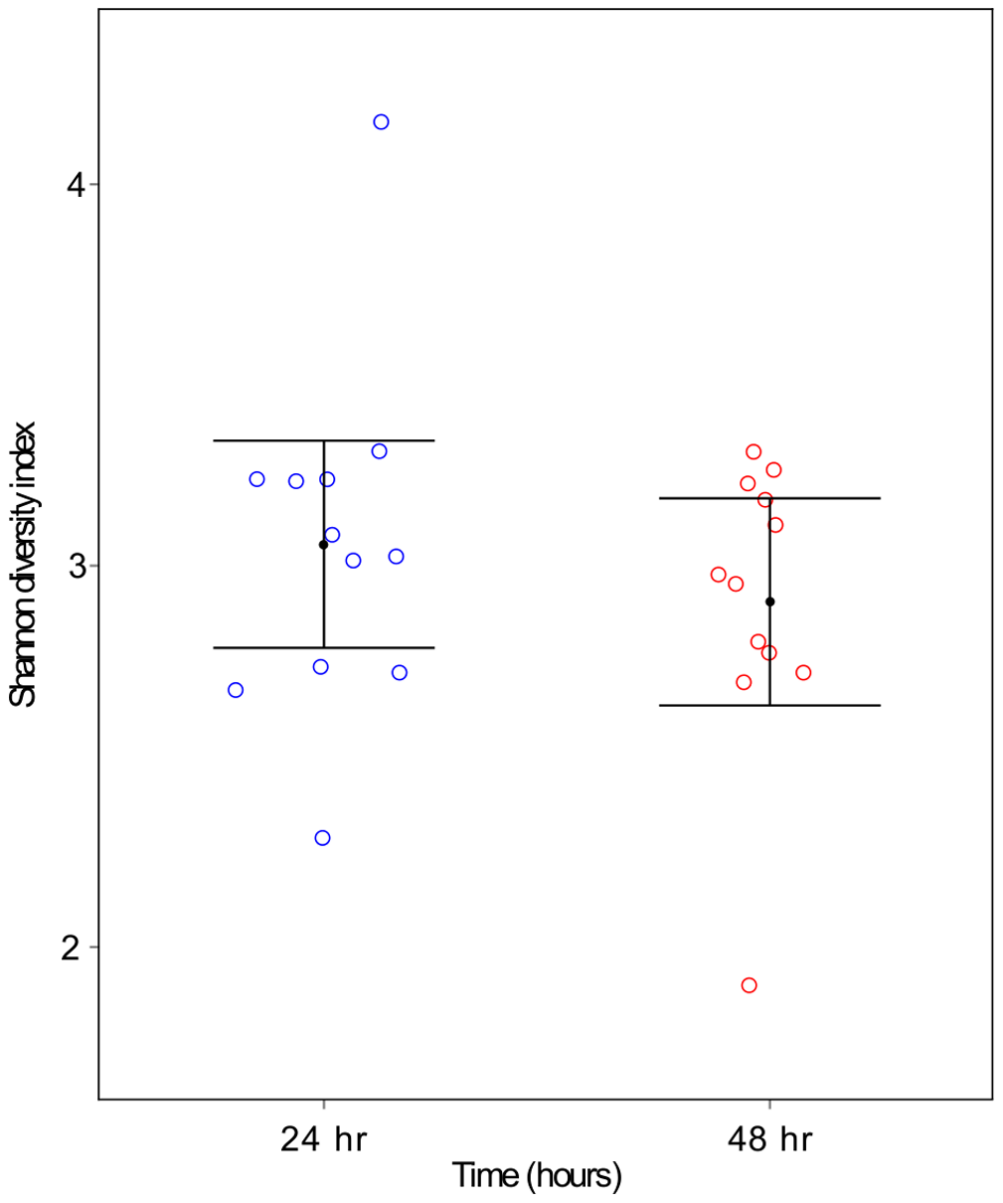
Shannon diversity index for supra-gingival plaque collected from dogs 24 hours (blue) and 48 hours (red) after a full mouth descale and polish. Error bars represent 95% confidence intervals around the mean.

**Figure 2:**
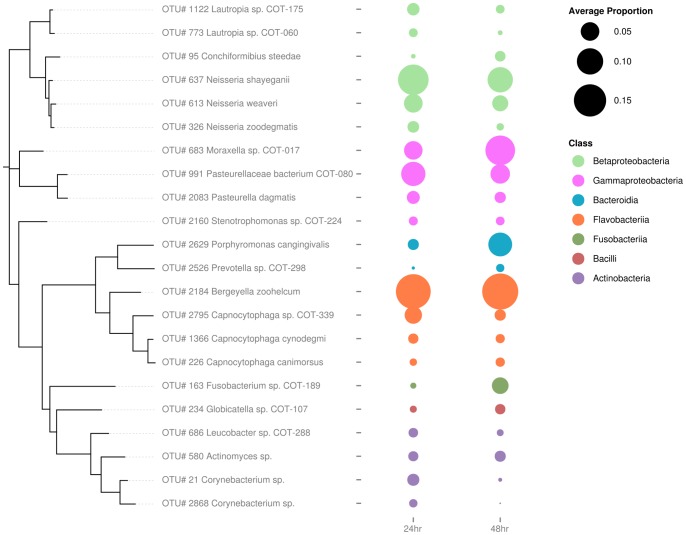
Relative abundances of the 22 dominant OTUs in sub-gingival plaque samples at 24 and 48 hours. Shown are all OTUs with at least 1% average abundance at either time point. OTUs were annotated against the Canine Oral Microbiome Database [10] and Silva SSU [22], [23] and a maximum likelihood tree was inferred to illustrate relatedness.

**Figure 3:**
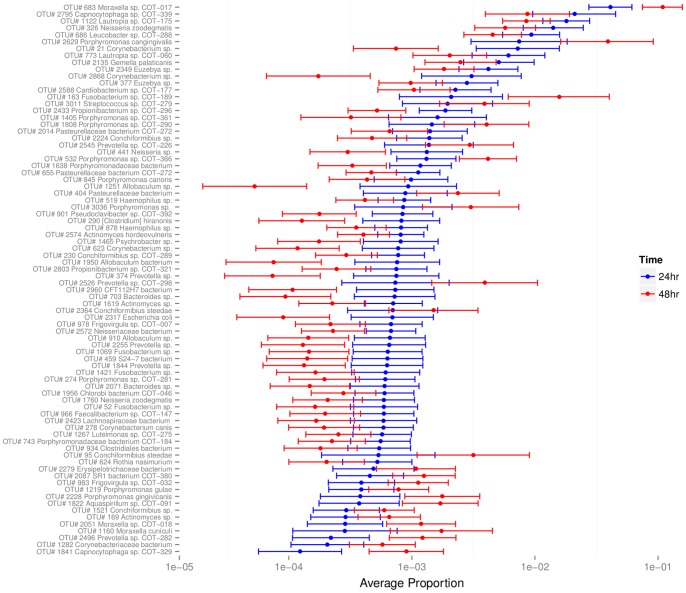
Mean proportions with 95% confidence intervals for OTUs with a significant time effect (>2-fold).

Analysis of the Shannon indices indicated that there was no significant difference between the diversity of the populations at the two sampling points (*p* = 0.292; Figure 1) with a difference of −0.15 from 24 hours to 48 hours with 95% confidence interval (-0.42, 0.12). Characterizing the changes over time for the OTUs which have high relative abundance (see Table 1) revealed that these OTUs were identified within the biofilm community at both time points (Figure 2). At both sampling points bacteria from the classes Gamaproteobacteteria, Betaproteobacteria and Flavobacteria dominated. In contrast, few members of the branches of the phylogenetic tree representing Fusobacteria and Bacilli were present; however, those that were found indicated a trend of increased relative abundance with time. Using GLMM analyses to investigate the time effect on each OTU individually resulted in 119 instances where significant effects were identified (BH, 5%). Of these, 73 OTUs were present at significantly higher levels at 24 hours than 48 hours while 46 fell into the reverse category and were present at significantly higher levels at 48 hours than 24 hours. OTUs with time effects equaling 2-fold or greater (total of 80 OTUs) are represented in Figure 3. Among the more abundant species two OTUs assigned to *Corynebacterium sp.* (OTU #21 and OTU #2868) had odds of presence within the community which decreased significantly over time. These were 9.88 and 17.62 times more likely to be found at 24 hours than 48 hours, respectively. Significant decreases in the odds of presence over time were exhibited by several members of the family *Neisseriaceae* (OTU #441 (4.40x) #326 (2.48x) #2572 (2.95x) #1760 (2.87x)). In contrast, *Moraxella sp.* COT-017 (OTU #683), one of the most abundant members of the biofilm community at both time points (Figure 2), was statistically 2.86 times more likely to be found at 48 hours. Furthermore, other *Moraxella* species, *Moraxella cuniculi* (OTU #1160) and *Moraxella sp.* COT-018 (OTU #2051), which were present at lower mean frequencies in the biofilm samples, were also statistically more likely to be found at 48 hours. Two other abundant OTUs *Fusobacterium sp.* COT-189 (OTU #163) and *Porphyromonas cangingivalis* (OTU #2629) exhibited the same trend being 7.67 and 5.51 times more likely to be part of the 48 hour rather than the 24 hour community, respectively.

### Primary colonization ability of canine oral bacteria *in vitro*

A set of canine oral isolates representative of the genera with high relative abundance in early supra-gingival plaque (see Table 1) were assessed for their ability to bind to the enamel substitute hydroxyapatite (HA). The initial primary colonization assay included visual scoring of the intensity of two stains, Crystal violet and Alamar blue, after bacterial isolates had interacted with the HA discs. The relative levels of intensity of each stain were ranked on a four point scale (Table 2). The three isolates with the maximum Crystal violet score all belonged to the genus *Neisseria* (*N. zoodegmatis* COT-349, *N. animaloris* COT-016 and *N. weaveri* COT-269). All had strong (+++) or moderate (++) levels of respiratory activity. *Stenotrophomonas sp.* COT-224 and *Corynebacterium sp.* 3105 showed moderate levels of biofilm formation and those biofilms had evidence of high respiratory activity. No evidence of primary colonization and subsequent biofilm formation was observed for *Actinomyces sp*. COT-083, *Moraxella sp.* COT-017 or *Pasteurella dagmatis* COT-092.

**Table 2:** Primary colonization of hydroxyapatite by canine oral bacterial isolates.

Species	Crystal violet stain intensity	Alamar blue stain intensity
Actinomyces canis	+	−
Actinomyces sp. COT-083	−	−
Bergeyella zoohelcum COT-186	+	+
Capnocytophaga sp. COT-339	+	+
Corynebacterium sp. (FJ374773)	++	+++
Moraxella sp. COT-017	−	−
Moraxella sp. COT-328	+	+
Neisseria animaloris COT-016	+++	+++
Neisseria shayeganii COT-090	+	+
Neisseria weaveri COT-269	+++	++
Neisseria zoodegmatis COT-349	+++	+++
Pasteurella dagmatis COT-092	−	−
Stenotrophomonas sp. COT-224	++	+++

Quantity (Crystal violet) and respiratory activity (Alamar blue) measured after 2 hours interaction with the surface followed by 24 hours growth.

Adhered bacteria stain dark purple with Crystal violet. The Alamar blue stain becomes pink in the presence of respiratory activity. Relative intensity of color change measured visually and ranked on a four point scale from most to least intense (+++, ++, +, -). Each score is the result of analysis of three biological replicates.

**Figure 4:**
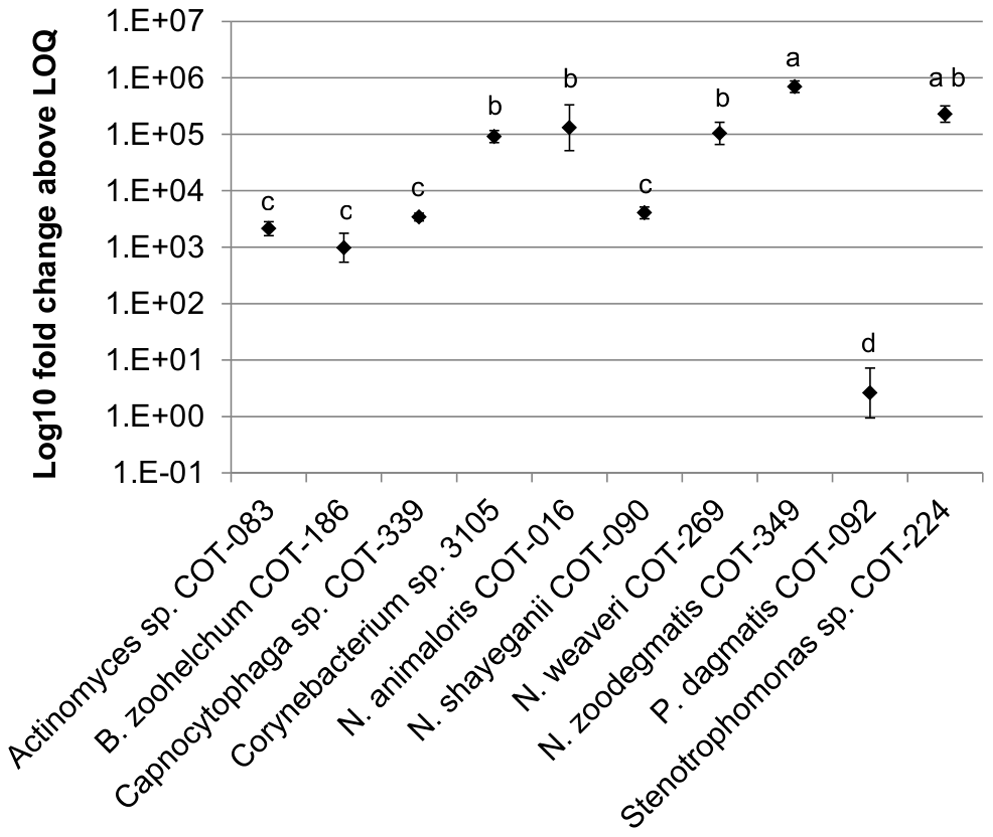
Primary colonization of hydroxyapatite by canine oral isolates. Quantitative PCR data analyzed using one way ANOVA of log_10_ fold change. Letters represent homogenous groupings by Tukey HSD at 5%. Error bars represent one standard deviation from the mean.

Molecular support for the visual characterization of primary colonization was obtained using species-specific qPCR assays (see Table S2; Figure 4). Fold change data indicated that the canine oral strains *N. zoodegmatis* COT-349, *N. animaloris* COT-016, *N. weaveri* COT-269, *Stenotrophomonas sp.* COT-224 and *Corynebacterium sp.* 3105 were all able to form significant biofilms on HA discs within a relatively short time frame (2 hours) (Table S3). It was confirmed by cell counts, (using biofilms grown in parallel), that the bacteria present on the discs were viable (data not shown).

### Building artificial early biofilm communities *in vitro*

To identify canine oral bacteria with potential importance as secondary biofilm colonizers, 11 individual isolates covering a broad spectrum of genera were tested in conjunction with two of the newly identified primary colonizers *N. zoodegmatis* COT-349 and *Corynebacterium sp*. 3105. These ‘bait’ species were chosen as representatives of strong and moderate primary colonization ability, respectively. Data in Tables 3 and 4 show the amount of each test species present in the resulting biofilm. In each experiment the amount of the primary colonizer species (*N. zoodegmatis* COT-349 or *Corynebacterium sp*. 3105) was confirmed as at least 10,000 fold above the LOQ (data not shown). Regardless of which pioneer species was used *Actinomyces canis* showed the highest level of incorporation into the biofilm. A fold increase of 698,629 (min 466,798, max 929,400) was measured with *N. zoodegmatis* COT-349 and 225,924 with *Corynebacterium sp.* 3105 (min 53,635, max 633,717). In addition, *Porphyromonas gingivicanis* COT-022, *Peptostreptococcaceae XI* [G-2] *sp.* COT-047 and *Moraxella sp.* COT-017 were all able to grow in a biofilm with *N. zoodegmatis* (Table 3) while *Moraxella sp.* COT-017, *Leucobacter sp*., *P. gingivicanis* COT-022 and *Pasteurellaceae sp.* COT-080 showed the potential to bind with *Corynebacterium sp*. 3105 (Table 4). Typically, species able to interact with the strong primary colonizer *N. zoodegmatis* COT-349 showed higher levels of growth (fold change) than those that interacted with *Corynebacterium sp*. 3105.

**Table 3:** Canine oral isolate secondary colonization potential measured by quantitative PCR with *Neisseria zoodegmatis* COT-349 present as primary colonizer.

				Range in fold change (minimum and maximum Cq)	
Strain	Mean Cq	Standard deviation of mean Cq	Mean fold change (relative to LOQ)	min	max
Actinomyces canis	16.41	0.52	698629	466798	929400
Bacteroides sp. COT-040	36.94	0.00	1	1	1
Bergeyella zoohelcum COT-186	35.47	0.00	1	1	1
Pasteurellaceae sp. COT-080	24.36	0.86	796	483	1536
Fusobacterium sp. COT-189	26.60	6.50	84	2	13421
Leucobacter sp.	25.99	2.40	569	144	3608
Moraxella sp. COT-017	21.90	1.48	17527	8143	56001
Peptostreptococcaceae XI [G-2] sp. COT-047	20.61	0.34	80871	63538	101849
Porphyromonas gingivicanis COT-022	18.58	1.00	87868	39773	143013
Porphyromonas sp. COT-108	27.04	4.72	148	16	6330
Xenophilus COT-264	29.32	2.10	36	33	134

Values are based on analysis of three biological replicates.

**Table 4:** Canine oral isolate secondary colonization potential measured by quantitative PCR with *Corynebacterium sp. 3105* present as primary colonizer.

				Range in fold change (minimum and maximum Cq)	
Strain	Mean Cq	Standard deviation of mean Cq	Mean fold change (relative to LOQ)	min	max
Actinomyces canis	18.03	1.85	225924	53635	633717
Bacteroides sp. COT-040	36.94	0.00	1	1	1
Bergeyella zoohelcum COT-186	35.47	0.00	1	1	1
Pasteurellaceae sp. COT-080	22.84	2.91	2288	222	7358
Fusobacterium sp. COT-189	24.80	1.40	295	98	601
Leucobacter sp.	22.94	1.09	4708	2255	10229
Moraxella sp. COT-017	22.95	1.97	8488	1753	20078
Peptostreptococcaceae XI [G-2] sp. COT-047	27.65	0.96	613	290	1032
Porphyromonas gingivicanis COT-022	23.56	2.13	2785	605	11566
Porphyromonas sp. COT-108	34.25	2.10	8	5	38
Xenophilus COT-264	24.47	1.65	947	485	3554

Values are based on analysis of three biological replicates.

**Figure 5:**
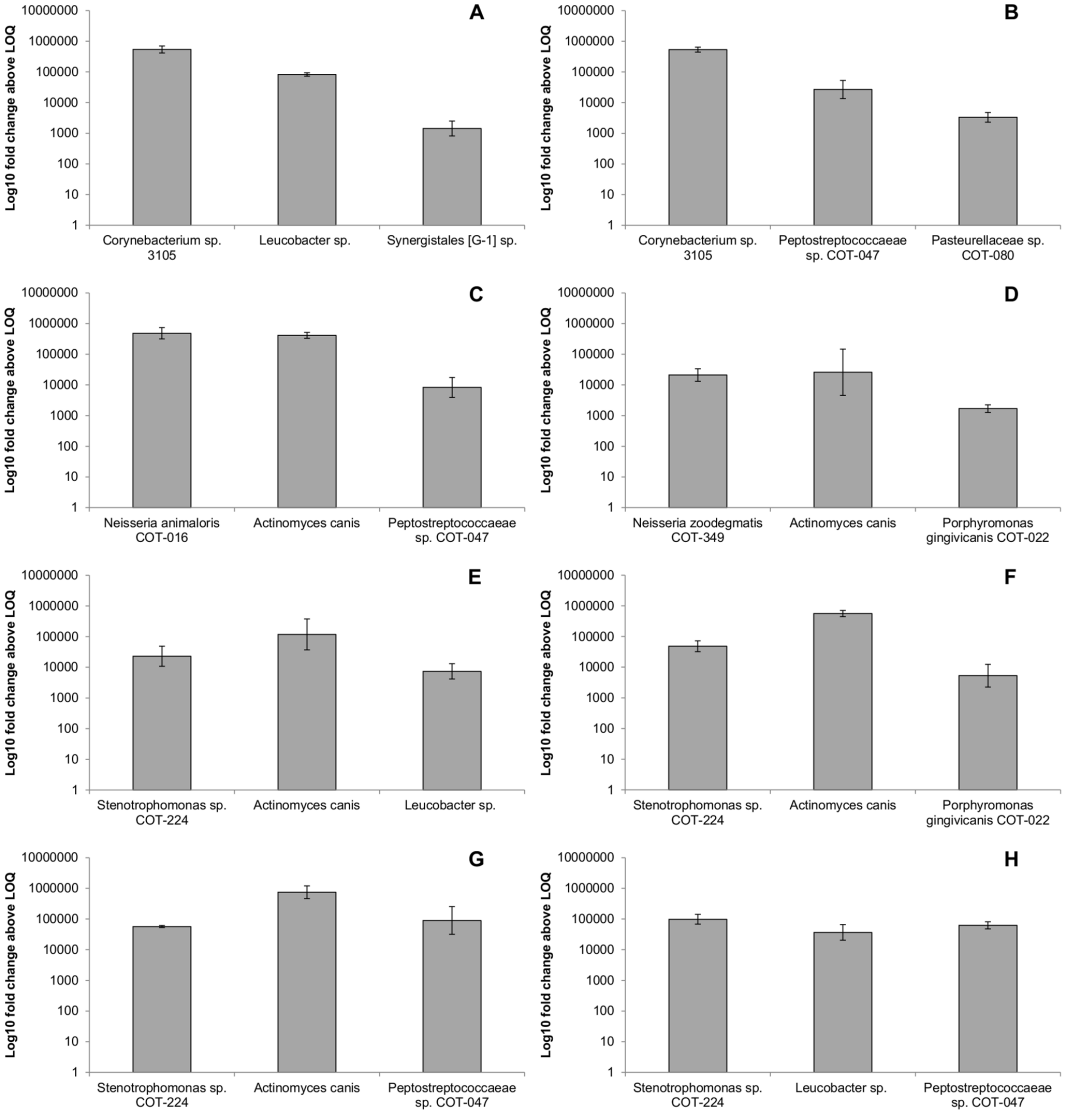
Viable 3-species bacterial biofilm communities. **A–B** *Corynebacterium sp.* 3015 primary colonizer. **C** *Neisseria animaloris* COT-016 primary colonizer. **D** *Neisseria zoodegmatis* COT-349 primary colonizer. **E–H** *Stenotrophomonas sp.* COT-224 primary colonizer. Amounts of each species measured using qPCR with species-specific assays. Error bars represent one standard deviation from the mean and are based on data from 3 biological replicates.

To gather further information about viable species combinations which may lead to the initiation and development of a canine plaque community a series of artificial 3-species biofilms were constructed and species growth analyzed using qPCR assays. The complete list of combinations is detailed in Table S4. A semi-arbitrary mean value of 1,000 fold above the LOQ of the assay was set as a threshold for confirmed biofilm formation. Four of the five primary colonizer species were tested in these communities: two representatives of the genus *Neisseria* (*N. zoodegmatis* COT-349 and *N. animaloris* COT-016) along with *Corynebacterium sp.* 3105 and *Stenotrophomonas sp.* COT-224. In total 8 of the 30 interactions passed the criteria for a functional 3-species community (Figure 5; Table S5). The species which featured most frequently in the role of third community member were *Peptostreptococcaceae sp.* COT-047, *Porphyromonas gingivicanis* COT-022 and *Leucobacter sp*.

## Discussion

This study is the first characterization of the bacteria important in early stage canine plaque biofilm development and begins to translate the growing bank of available knowledge on canine oral microbiota into an understanding of the dynamic processes occurring during plaque formation. Culture-independent sequencing of plaque forming on freshly polished teeth has provided, to our knowledge, the first accurate representation of the early *in vivo* colonization process in dogs. The information gathered from the natural setting, in conjunction with a suite of defined canine oral isolates and validated species-specific qPCR probes, enabled the re-creation of viable early biofilm communities *in vitro*.

*In vivo* supra-gingival plaque was found to be dominated by a relatively small number of taxa with 17 OTUs accounting for, on average, over 70% of sequence reads. Furthermore, just two genera (*Bergeyella* and *Neisseria*) accounted for a third of the biofilm community. In comparison, pyrosequencing (to a similar depth) of established sub-gingival plaque from a large cohort of dogs in a separate study revealed that the 26 most abundant species accounted for approximately 50% of the sequence reads in these more mature communities [11]. It is evident that, with 134 OTUs (plus ‘rares’) meeting our analysis criteria, the biofilm community is already well established 24 hours after professional cleaning. When making comparisons between our early biofilm pyrosequencing data and that from other studies it is important to consider the type of plaque collected, i.e. sub-gingival or supra-gingival. Current understanding suggests that supra-gingival plaque is typically less well developed and contains fewer disease-associated species than sub-gingival plaque [33], [34]. A recent time course study of early human plaque development *in vivo* using a checkerboard DNA-DNA hybridization approach revealed that sub-gingival plaque tends to exhibit a slower and different re-development when compared to supra-gingival plaque [35]. This highlights that the different micro-environments encountered at the two sites may provide the opportunity for communities to initiate and evolve down different routes.

The HA disc biofilm model used filter-sterilized canine saliva with 38°C incubation to provide a close representation of the natural growth setting. Biofilm formation was then assessed via a combination of visual and molecular tools. Presence of a species in qPCR experiments was defined using a measure of log_10_ fold change relative to the assay LOQ. While this approach does not allow accurate comparisons between amounts of each species, such as would be possible using an absolute quantification with conversion to genome equivalents, it can clearly indicate presence or absence of growth. Five of the isolates tested in the *in vitro* system had a clear ability to form viable, growing biofilms on the disc surface. These included three members of the genus *Neisseria* (*N. animaloris* COT-016, *N. zoodegmatis* COT-349, and *N. weaveri* COT-269) along with *Corynebacterium sp.* 3105 and *Stenotrophomonas sp.* COT-224. In a previous study of sub-gingival plaque, *N. animaloris* was most prevalent in samples from dogs with gingivitis [11]. This particular *Neisseria* species was not among the most prevalent in early biofilms in this study suggesting that although it has the ability to colonize enamel it may be more commonly associated with a later stage of biofilm development. Each of the other species had previously been identified as associated with a healthy oral environment [11] and all prefer an oxygen-rich atmosphere. These characteristics fit the typical profile of a pioneer species [36]. Interestingly, aside from *Corynebacterium sp.* 3105, all are Gram negative bacteria, supporting the idea that the healthy/early biofilm community in dogs exhibits the opposite trend to human plaque [11]. Of the 17 most prevalent species in Table 1, 14 are Gram negative, and for the OTUs present at >1%, Gram negative species account for 94% of the sequence reads. It is important to consider that DNA extraction methods can lead to the introduction of bias in pyrosequencing data. In this study two strategies were employed to try to combat this; a lysis method specifically designed to improve recovery of Gram positive bacteria and the inclusion of additional primers during 16S rDNA amplification to increase the potential to detect members of the genus *Bifidobacter*. Although the representation of Gram positive species observed in early plaque biofilms was low, when the same methodology was used to characterize plaque samples from a large cohort of dogs similarly low levels of Gram positive bacteria were found in healthy plaque, while significantly higher numbers were present in disease-associated plaque (11). Analysis of the colonization of enamel chips worn in the human mouth using a 16S rDNA cloning approach revealed that small time-dependent shifts in community composition occur during the 4–8 hour stage of biofilm development [12]. Typically these shifts were characterized by a redistribution of the various streptococci present, nonetheless, inter-individual variation between the three subjects studied suggested that the ecological shifts and changes in biofilm diversity were strongly subject-dependent. In canine plaque we found no difference in the overall diversity of biofilms between 24 hour and 48 hour time points, despite this a considerable number of statistically significant changes in the proportions of individual OTUs were detected. These included lower odds of *Neisseria* and *Corynebacterium* species being present over time, a trend which supports the proposed role of these bacteria as primary colonizers of enamel which are lost from the community once their role is complete.

In a recent *in vitro* study of early human biofilm communities, removal of all known primary colonizers from the pool of experimental species still resulted in the formation of a plaque community which reached normal thickness and bacterial load but exhibited a different physical structure and ecological succession [37]. The majority of work on human plaque reveals a dominance of streptococci early in plaque development and members of the genus are championed as the key primary colonizers of enamel. More recently, evidence from sequence-based *in vivo* studies has indicated that *Neisseria* species also play a role [35], [38]–[40]. Given the lack of streptococci in dog plaque it is plausible that the streptococcal niche is instead occupied by *Neisseria* species. The pH of dog saliva is much more alkaline (pH 8.5 [41]) than human saliva making it less favorable to acidogenic streptococci. The difference in the amount of sugar present in the average diets of most humans and dogs would also support this idea. A recent study of calcified dental plaque on ancient teeth revealed two distinct shifts in the human oral microbiome driven by diet. In Neolithic times a change from a hunter-gather lifestyle to farming induced a change to disease-like plaque configurations. This was followed more recently by the appearance of cariogenic populations resulting from an increase in processed sugar consumption after the industrial revolution [42]. Although *Neisseria* species are among the most prevalent species in *in vivo* early canine biofilms perhaps the first time point investigated here (24 hours) is already too late to capture their full role in primary colonization.

The projected role for *Stenotrophomonas* species as strong primary colonizers would not be predicted based on analysis of only the *in vivo* data. The OTU mapping to *Stenotrophomonas sp.* COT-224 has a relative abundance of 1.13%, and no significant time-dependent effects were identified. Perhaps by the first sampling point at 24 hours it has completed its role in the community and been replaced. Alternatively it may have merely been outcompeted by *Neisseria* species, with the laboratory model reflecting a potential rather than an actual role.

Important roles for *Moraxella*, *Porphyromonas*, *Pasteurella,* and *Actinomyces* species during nascent biofilm development were also identified. *Moraxella sp.* COT-017 was the third most abundant species across both time points. It was 2.86 times more likely to be present at 48 hours than 24 hours and showed good evidence of incorporation at the second level of colonization *in vitro*; notwithstanding this, a viable 3-species community involving this species has yet to be confirmed. In a large cross-sectional study of canine plaque *Porphyromonas cangingivalis* was found to be the most common bacterial species across all health states with a relative abundance of 7.4% in sub-gingival plaque [11]. In early supra-gingival plaque samples, the levels of *P. cangingivalis* were seen to increase significantly between 24 hours and 48 hours, consistent with its incorporation once the community starts to mature and conditions become more favorable to its anaerobic lifestyle. The high degree of incorporation of *Actinomyces canis* as a secondary colonizer into biofilms formed using each of the primary colonizers is interesting, given that it has been shown to co-aggregate prolifically in plaque [16] and is associated with gingivitis or early periodontitis. Fluorescent *in situ* hybridization (FISH) studies of adhesion on pristine tooth surfaces have identified patchy co-localized distributions of human oral *Streptococcus* and *Actinomyces* species [43]; a similar type of aggregation may occur in canine plaque with *Actinomyces* interacting with *Neisseria* or *Stenotrophomonas* species in saliva before binding to enamel. One hypothesis could be that binding of *Actinomyces* species represents an important turning point for the biofilm, altering the status quo and providing possibilities for a move towards a more disease-associated community through binding interactions.

Additionally the *in vivo* sequencing data hints at a role for *Fusobacterium sp.* COT-189 in the developing biofilm as it was one of the species that showed the biggest increase from 24 to 48 hours (7.63x – Figure 2). This is particularly interesting as it parallels the current understanding of human plaque succession in which *Fusobacterium nucleatum* has been implicated as a bridging species between early and late colonizers that allows the development of disease-like biofilm conditions [44]–[46].

One of the most interesting and potentially important, results from this study is the paradox between the high relative abundance of *Bergeyella zoohelcum* COT-186 in the *in vivo* samples (16.97%) and its lack of integration into artificial biofilms. In support of the data reported here *B. zoohelcum* was identified as the third most highly abundant species (5.48%), after *Porphyromonas cangingivalis* (10.47%) and *Moraxella sp.* COT-396 (6.61%), in a study of healthy canine sub-gingival plaque samples from a cross-section of breeds [11]. Also, in a separate analysis of plaque from 6 dogs [9], *Bergeyella* was among the most prevalent taxa representing 2.7% of sequence reads. It is possible that the laboratory isolate used in this study is not representative of environmental strains. To account for this a second isolate was tested. Despite having 100% 16S sequence identity to *B. zoohelcum* COT-186 it also was unable to act as a primary colonizer of enamel. Other possible explanations for the discrepancy include reliance on a specific metabolic association with another species, or requirement for a host factor not present in the filtered canine saliva used as a source of growth nutrients.

**Figure 6:**
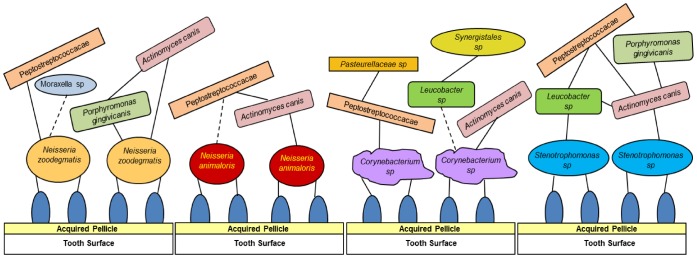
Hypothesized spatio-temporal model of *in vitro* early canine oral biofilm communities. Interaction networks for four primary colonizer species *Neisseria animaloris* COT-016, *Neisseria zoodegmati*s COT-349, *Corynebacterium sp.* 3105 and *Stenotrophomonas sp*. COT-224. Each community was identified using canine oral isolates and species-specific qPCR probes. Dotted lines represent tentative interactions.

While only selected combinations of species were tested *in vitro* (the goal being to identify a small number of early biofilm communities rather than to understand the myriad of possible interactions) differences in the directions of the paths which the biofilm may take depending on the initial colonizing species are already evident. Taken together the data generated from the *in vivo* and *in vitro* studies have led us to postulate four potential models of early biofilm formation (Figure 6). Each of the model biofilms depicted comprises a pioneer bacterial species, which is able to bind to saliva-coated enamel, and a series of additional species which may interact to create a community. Although the interactions outlined are described as binding interactions it becomes difficult beyond the primary colonization step to describe how interactions are manifested. For some of the bacteria described co-aggregation data [16] lends support for a physical interaction process. However, to be able to define situations as true binding, rather than as associations, an analysis of the bacterial surface receptors would be required. Our evidence indicates colonization of HA is underway within 2 hours and small communities are established within 4 hours of inoculation. More work will be required to gain a full temporal understanding of community development.

Growing evidence suggests that the oral ecosystem is a tripartite system with microorganisms, host and environment all contributing to plaque maturation and the onset of periodontal disease [47]. This study represents a first step in understanding how bacterial interactions can lead to canine plaque formation, combining observations made both *in vivo* and *in vitro* to describe early biofilm communities. Significant differences have been highlighted between human and canine oral environments indicating that knowledge about plaque in one species cannot be directly transferred to the other. A more detailed knowledge of the key biofilm bacteria and the mechanisms through which they act will enable future characterization of the processes which lead to canine periodontal disease.

## Supporting Information

Table S1 **List of bacteria used in the study.**
(DOCX)

Table S2 **qPCR assay details.**
(DOCX)

Table S3 **qPCR data associated with primary colonization.**
(DOCX)

Table S4 **List of artificial 3-species communities tested** ***in vitro*** **.**
(DOCX)

Table S5 **qPCR data associated with artificial 3-species communities.**
(DOCX)
