# Supplementary material for: Early Canine Plaque Biofilms: Characterization of Key Bacterial Interactions Involved in Initial Colonization of Enamel
Source: PLoS One. 2014 Dec 2;9(12):e113744. doi: 10.1371/journal.pone.0113744 (PMC4252054; doi:10.1371/journal.pone.0113744)
Supplement: Table S1 — List of bacteria used in the study. (DOCX) [file pone.0113744.s001.docx]

| Name | COT # or qPCR assay name | Health state association | Preferred atmosphere for growth | Gram status |
| --- | --- | --- | --- | --- |
| Actinomyces_canis | A. canis | Gingivitis/PD1 | MiO2 | Positive |
| Actinomyces_sp. | COT-083 | PD1 | O2 | Positive |
| Bacteroides sp. | COT-040 | Health | AnO2 | Negative |
| Bergeyella zoohelcum | COT-186 | Health | O2 | Negative |
| Capnocytophaga sp. | COT-339 | Health | O2 | Negative |
| Corynebacterium sp. 3105 | FJ374773 | Health/Gingivitis | O2 | Positive |
| Fusobacterium sp. | COT-189 | Health | AnO2 | Negative |
| Leucobacter sp | AY827913 | None | O2 | Negative |
| Moraxella sp. | COT-017 | Health | MiO2 | Negative |
| Moraxella sp. | COT-328 | Health | O2 | Negative |
| Neisseria animaloris | COT-016 | Gingivitis | O2 | Negative |
| Neisseria shayeganii | COT-090 | Health | MiO2 | Negative |
| Neisseria zoodegmatis | COT-349 | Health | O2 | Negative |
| Neisseria weaveri | COT-269 | Health | O2 | Negative |
| Pasteurella dagmatis | COT-092 | Health/Gingivitis | O2 | Negative |
| Pasteurellaceae sp. | COT-080 | Health | O2 | Negative |
| Peptostreptococcaceae XI [G-2] sp. | COT-047 | None | AnO2 | Positive |
| Porphyromonas gingivicanis | COT-022 | None | AnO2 | Negative |
| Porphyromonas sp. | COT-108 | Gingivitis/PD1 | AnO2 | Negative |
| Synergistales sp. | COT-178 | PD1 | AnO2 | Negative |
| Stenotrophomonas sp. | COT-224 | Health | O2 | Negative |
| Xenophilus sp. | COT-264 | Health | O2 | Negative |
