# Supplementary material for: Early Canine Plaque Biofilms: Characterization of Key Bacterial Interactions Involved in Initial Colonization of Enamel
Source: PLoS One. 2014 Dec 2;9(12):e113744. doi: 10.1371/journal.pone.0113744 (PMC4252054; doi:10.1371/journal.pone.0113744)
Supplement: Table S2 — qPCR assay details. (DOCX) [file pone.0113744.s002.docx]

| Assay name | Target Sequence | Forward primer | Probe | Reverse primer | LOQ |
| --- | --- | --- | --- | --- | --- |
| COT016 | Neisseria sp. | AACTGTCCGAAAGGATGGCTAA | ATATTCTCTGAGGAGGAAAG | CGCAAGGCCCGAAGGT | 35.00 |
| COT017 | Moraxella sp. | ACAACATTCCGAAAGGAATGC | AGCGCAAGGTCMRAMGATCCCCTGCTTT | CTTTACCCCACCAACTAGCT | 36.00 |
| COT022 | Porphyromonas gingivicanis | GATAGGCTTAACACATGCAAGTC | CAGCATAATTTTAGCTTGCTAAGATTGATGGCGAC | ACCGAGCTATCCCCTAATTACA | 35.00 |
| COT040 | Bacteroides sp. | AAGATGGATTAATACTTGATGGTATATAC | ACGCATCCCCATCTATCACCGATAAATCTT | ACCCCTAACCATCGAAGTCTT | 36.94 |
| COT047 | Peptostreptococcaceae XI [G-2] sp. | GGAAGTTTTCGGATGGAAGAAAAATTACT | CGTTACTCACCCGTCCGCCACT | ATATTTATATCTCCCGATATACTTACTTC | 36.91 |
| COT080 | Pasteurellaceae sp. | CCTTCGGGTTGTAAAGTTCTT | AAGGTATCAACTWTAATAGAGTTGGTAAWDGACGTTAT | TGCTGGCACGGAGTTAG | 34.00 |
| COT083 | Actinomyces sp. | GGGGCTTTTGCTTTGTTGGT | CCTCAACATTTGAATAACTCCATGAAAGTGGGGC | CAACCACCATGCGATGACTG | 34.00 |
| COT 090/349 | Neisseria sp. (N. shayeganii/N. zoodegmatis) | GGACGGCAGCRCARAGAAG | TCGCCACTCGCCACCCAAGAAGC | GTTATCCCCCACTACTCGGTAC | 35.81 |
| COT 092 | Pasteurella dagmatis | CGTAKTATCGAGAGATGAAAGGGT | CACCAACTACCTAATCCCACTTGGGCTCAT | CCTCTCAGACCAGCTAGAGATCG | 34.94 |
| COT 108 | Porphyromonas sp. | ATGGCGACCGGCGGAT | CCCCTCTGACAGGTAAGTTGCATACGC | CTTGAAATACCATGCAGYATCTCAAG | 34.25 |
| COT 178 | Synergistales sp. | AGTACAACATGAGAAGCTGTCCAATAG | TCGGAAACGGACCCTAATACCCCA | CATCGCTGCTTTAACCAAAAGG | 31.30 |
| COT 186 | Bergeyella sp. | GGGATTTGTTGGTTAGCTTGCT | ACTGATAATGAGAGCGGC | GGCAGGTTGCACACGTGTTA | 35.47 |
| COT 189 | Fusobacterium sp. | GAACGCTGACAGAATGCTTAAC | CGCCACCCAAACCGAAGTTCAAGTTGA | CCGTTTCCAGATGTTGTCCYRG | 33.00 |
| COT224 | Stenotrophomonas sp. | GGATAACGTAGGGAAACTTACGC | CCGAAGGTCCCCTGCTTTCTCCCGTAG | CCCCAACTAGCTAATCCGACG | 35.00 |
| COT264 | Xenophilus sp. | AGCGCGGGCTTCGGC | CACCCGTTCGCCACTCGCCGC | CCCCTGCTTTCATCCGTAGATC | 34.36 |
| COT 339 | Capnocytophaga sp. | CAGGCCTAACACATGCAAGTC | CGGTCTCAAACTACTAAAGTAGCTCTACCCCT | ACTATGAGGTATTAATCCAAATTTCTCTG | 35.00 |
| A.Canis | Actinomyces canis | GAAACRGATGCTAATACCGGG | CCCCACACCATGCGAYRYGAGGTA | GCCATCACCCCACCAACA | 35.82 |
| AY827913 | Leucobacter sp. | TAACCTGCCCTCTACTCTGG | TGTCTAATACTGGATACGACCTTGAACCGCA | AAAAAACTTTCCACCAAGAAACC | 35.14 |
| FJ374773 | Corynebacterium sp. 3105 | GCTTGGGAAACTGGGTCTAATAC | TCCACCACCAACACTAAATGATGGTCCTA | GCTCATCTCGCACCGAAAA | 36.11 |

LOQ refers to the Limit of Quantification of the assay.
