# Supplementary material for: Early Canine Plaque Biofilms: Characterization of Key Bacterial Interactions Involved in Initial Colonization of Enamel
Source: PLoS One. 2014 Dec 2;9(12):e113744. doi: 10.1371/journal.pone.0113744 (PMC4252054; doi:10.1371/journal.pone.0113744)
Supplement: Table S3 — qPCR data associated with primary colonization. (DOCX) [file pone.0113744.s003.docx]

| Bacterial Species | Mean Cq | Standard Deviation of Mean Cq | Assay LOQ |
| --- | --- | --- | --- |
| Actinomyces sp. COT-083 | 22.95 | 0.41 | 34.00 |
| B. zoohelchum COT-186 | 25.54 | 0.85 | 35.47 |
| Capnocytophaga sp. COT-339 | 23.26 | 0.23 | 35.00 |
| Corynebacterium sp. 3105 | 19.64 | 0.35 | 36.11 |
| N. animaloris COT-016 | 18.01 | 1.35 | 35.00 |
| N. shayeganii COT-090 | 23.82 | 0.34 | 35.81 |
| N. weaveri COT-269 | 19.14 | 0.66 | 35.81 |
| N. zoodegmatis COT-349 | 16.40 | 0.33 | 35.81 |
| P. dagmatis COT-092 | 33.56 | 1.46 | 34.94 |
| Stenotrophomonas sp. COT-224 | 17.20 | 0.48 | 35.00 |
