# Supplementary material for: Early Canine Plaque Biofilms: Characterization of Key Bacterial Interactions Involved in Initial Colonization of Enamel
Source: PLoS One. 2014 Dec 2;9(12):e113744. doi: 10.1371/journal.pone.0113744 (PMC4252054; doi:10.1371/journal.pone.0113744)
Supplement: Table S4 — List of artificial 3-species communities tested in vitro . (DOCX) [file pone.0113744.s004.docx]

| Species 1 (Primary) | Species 2 | Species 3 |
| --- | --- | --- |
| Corynebacterium sp. 3105 | Actinomyces canis | Synergistales [G-1] sp. COT-178 |
| Corynebacterium sp. 3105 | Actinomyces canis | Moraxella sp. COT-017 |
| Corynebacterium sp. 3105 | Actinomyces canis | Pasteurellaceae sp. COT-080 |
| Corynebacterium sp. 3105 | Actinomyces canis | Peptostreptococcaceae sp. COT-047 |
| Corynebacterium sp. 3105 | Moraxella sp. COT-017 | Pasteurellaceae sp. COT-080 |
| Corynebacterium sp. 3105 | Leucobacter sp. | Synergistales [G-1] sp. COT-178 |
| Corynebacterium sp. 3105 | Leucobacter sp. | Pasteurella dagmatis COT-092 |
| Corynebacterium sp. 3105 | Pasteurellaceae sp. COT-080 | Peptostreptococcaceae sp. COT-047 |
| Corynebacterium sp. 3105 | Pasteurellaceae sp. COT-080 | Fusobacterium sp. COT-189 |
| Neisseria animaloris COT-016 | Actinomyces canis | Porphyromonas gingivicanis COT-022 |
| Neisseria animaloris COT-016 | Actinomyces canis | Moraxella sp. COT-017 |
| Neisseria animaloris COT-016 | Actinomyces canis | Peptostreptococcaceae sp. COT-047 |
| Neisseria animaloris COT-016 | Actinomyces canis | Pasteurellaceae sp. COT-080 |
| Neisseria animaloris COT-016 | Moraxella sp. COT-017 | Pasteurellaceae sp. COT-080 |
| Neisseria zoodegmatis COT-349 | Actinomyces canis | Porphyromonas gingivicanis COT-022 |
| Neisseria zoodegmatis COT-349 | Actinomyces canis | Moraxella sp. COT-017 |
| Neisseria zoodegmatis COT-349 | Actinomyces canis | Peptostreptococcaceae sp. COT-047 |
| Neisseria zoodegmatis COT-349 | Actinomyces canis | Pasteurellaceae sp. COT-080 |
| Neisseria zoodegmatis COT-349 | Peptostreptococcaceae sp. COT-047 | Moraxella sp. COT-017 |
| Neisseria zoodegmatis COT-349 | Peptostreptococcaceae sp. COT-047 | Pasteurellaceae sp. COT-080 |
| Neisseria zoodegmatis COT-349 | Peptostreptococcaceae sp. COT-047 | Fusobacterium sp. COT-189 |
| Neisseria zoodegmatis COT-349 | Pasteurellaceae sp. COT-080 | Fusobacterium sp. COT-189 |
| Stenotrophomonas sp. COT-224 | Actinomyces canis | Leucobacter sp. |
| Stenotrophomonas sp. COT-224 | Actinomyces canis | Pasteurellaceae sp. COT-080 |
| Stenotrophomonas sp. COT-224 | Actinomyces canis | Porphyromonas gingivicanis COT-022 |
| Stenotrophomonas sp. COT-224 | Actinomyces canis | Peptostreptococcaceae sp. COT-047 |
| Stenotrophomonas sp. COT-224 | Actinomyces canis | Moraxella sp. COT-017 |
| Stenotrophomonas sp. COT-224 | Leucobacter sp. | Peptostreptococcaceae sp. COT-047 |
| Stenotrophomonas sp. COT-224 | Peptostreptococcaceae sp. COT-047 | Moraxella sp. COT-017 |
| Stenotrophomonas sp. COT-224 | Pasteurellaceae sp. COT-080 | Porphyromonas gingivicanis COT-022 |
