# Supplementary material for: Early Canine Plaque Biofilms: Characterization of Key Bacterial Interactions Involved in Initial Colonization of Enamel
Source: PLoS One. 2014 Dec 2;9(12):e113744. doi: 10.1371/journal.pone.0113744 (PMC4252054; doi:10.1371/journal.pone.0113744)
Supplement: Table S5 — qPCR data associated with artificial 3-species communities. (DOCX) [file pone.0113744.s005.docx]

| Bacterial Species | Mean Cq | Standard Deviation of Mean Cq | Assay LOQ |
| --- | --- | --- | --- |
| Community 1 |  |  |  |
| Corynebacterium sp. 3105 | 17.07 | 0.38 | 36.11 |
| Leucobacter sp. | 18.81 | 0.19 | 35.14 |
| Synergistales [G-1] sp. | 20.82 | 0.80 | 31.30 |
| Community 2 |  |  |  |
| Corynebacterium sp. 3105 | 17.08 | 0.27 | 36.11 |
| Peptostreptococcaeae sp. COT-047 | 22.20 | 0.98 | 36.91 |
| Pasteurellaceae sp. COT-080 | 22.30 | 0.52 | 34.00 |
| Community 3 |  |  |  |
| Neisseria animaloris COT-016 | 16.12 | 0.61 | 35.00 |
| Actinomyces canis | 17.17 | 0.31 | 35.82 |
| Peptostreptococcaeae sp. COT-047 | 23.89 | 1.08 | 36.91 |
| Community 4 |  |  |  |
| Neisseria zoodegmatis COT-349 | 21.45 | 0.68 | 35.81 |
| Actinomyces canis | 21.15 | 2.50 | 35.82 |
| Porphyromonas gingivicanis COT-022 | 24.28 | 0.42 | 35.00 |
| Community 5 |  |  |  |
| Stenotrophomonas sp. COT-224 | 20.52 | 1.09 | 35.00 |
| Actinomyces canis | 18.97 | 1.68 | 35.82 |
| Leucobacter sp. | 22.29 | 0.83 | 35.14 |
| Community 6 |  |  |  |
| Stenotrophomonas sp. COT-224 | 19.44 | 0.58 | 35.00 |
| Actinomyces canis | 16.73 | 0.33 | 35.82 |
| Porphyromonas gingivicanis COT-022 | 22.63 | 1.23 | 35.00 |
| Community 7 |  |  |  |
| Stenotrophomonas sp. COT-224 | 19.20 | 0.11 | 35.00 |
| Actinomyces canis | 16.31 | 0.68 | 35.82 |
| Peptostreptococcaeae sp. COT-047 | 20.46 | 1.49 | 36.91 |
| Community 8 |  |  |  |
| Stenotrophomonas sp. COT-224 | 18.42 | 0.53 | 35.00 |
| Leucobacter sp. | 19.98 | 0.85 | 35.14 |
| Peptostreptococcaeae sp. COT-047 | 20.99 | 0.38 | 36.91 |
